# Treating Stage IV periodontitis: the UK adolopment of the EFP Stage IV Periodontitis Treatment Guidelines

**DOI:** 10.1038/s41415-026-9887-0

**Published:** 2026-09-11

**Authors:** Devan S. Raindi, Moritz Kebschull, Iain Chapple

**Affiliations:** 835120895802490872866 Scott Arms Dental Practice, Birmingham, United Kingdom; 093830625726842116329 NIHR BRC Birmingham, University of Birmingham, United Kingdom

## Abstract

The effects and treatment of periodontitis go beyond the inflammatory lesion of the periodontium, with sequelae of Stage IV and sometimes Stage III disease requiring rehabilitation of the dentition. In October 2022, the British Society of Periodontology carried out an adolopment process (in a similar fashion to the Stage I–III guideline) for the European Federation of Periodontology's S3 Clinical Guideline for the Treatment of Stage IV Periodontitis. This article covers the structure of an S3-level guideline in general and the adoloped recommendations across all case types for this specific guideline document.

## Key points

- An S3-level guideline forms the highest level of guideline development combining both a comprehensive review of the evidence and a formalised consensus process.
- Tooth retention remains the first-line treatment strategy wherever feasible in the overall management of Stage IV periodontitis albeit a range of restorative options are available based on the patient's clinical presentation.
- Supportive periodontal care is an essential part of a periodontitis patient's treatment plan and should be tailored to the patient's individual needs.

## Introduction

The effects and treatment of periodontitis go beyond the inflammatory lesion within the periodontium with sequelae of Stage IV and sometimes Stage III disease requiring rehabilitation of the dentition.^1^ There is also a growing evidence base confirming an influence of periodontitis and its treatment on systemic health.^2^^,^^3^

Before proceeding, it is important to note that Stage IV periodontitis in the 2017 World Workshop Classification (published as the 2018 Classification) of periodontal and peri-implant diseases and conditions^4^ refers to the complications of periodontitis and restoring a patient to a functional dentition, whereas following the British Society of Periodontology (BSP) Implementation of the 2018 Classification, Stage IV periodontitis within the BSP's implementation of the 2018 Classification is defined by advanced bone loss within the apical third of teeth.^5^ The latter will in almost all cases result in tooth movement and complications that require rehabilitation; therefore, the Stage IV guideline applies equally to the BSP implementation of the classification. Those complicating factors from the original classification include:

- Tooth loss ≥5 teeth due to periodontitis
- Masticatory dysfunction
- Secondary occlusal trauma
- Severe ridge defects
- Bite collapse, drifting, flaring
- Less than 20 remaining teeth (ten opposing pairs).

Clearly, most, if not all, of these complications will also be present in BSP Stage IV periodontitis patients. The guideline is highly relevant to general dental practitioners as, even if a specialist team is involved in managing periodontitis, rehabilitation often takes place within a general dental setting and should involve an evidence-based approach. It is also likely that patients will see their general dental teams as part of a longer-term supportive care plan.

The aim of this article is to provide an overview of the European Federation of Periodontology (EFP) S3 Clinical Guideline for the Treatment of Stage IV Periodontitis, initially published in 2022, summarising the key recommendations for UK practitioners, subsequently published following an adolopment process in 2025.^1^^,^^6^

## Understanding the S3 guidelines

The process involved in arriving at the final guideline document for Stage IV periodontitis followed a similar structure to that involved in the Stages I–III periodontitis clinical guideline.

When guidelines are proposed for clinical care, there are various ways in which they can be developed – namely: an informal recommendation by a group of experts; a structured consensus; the systematic evaluation of the evidence; or ideally, a combination of both these, i.e., synthesis of available evidence and structured consensus by a representative group. Table 1 summarises the different levels of guideline development and as can clearly be seen, S3 guideline development represents the highest level.

**Table 1:** Levels of guidance development (Association of the Scientific Medical Societies in Germany – Standing Guidelines Commission, 2023)^7^

**S-classification**		**Description**
S3	Evidence and consensus-based guidelines	Representative committee, systematic review and synthesis of evidence, structured consensus process
S2e	Evidence-based guideline	Systematic review and synthesis of evidence
S2k	Consensus-based guideline	Representative committee and structured consensus process
S1	Recommendation by group of experts	Consensus through informal expert opinion

As part of the evidence-based approach to guideline development, invited systematic reviewers submitted specific PICO(S) questions for consideration by the steering committee. The resulting 13 systematic reviews were proposed to working group chairs and methodological consultants for approval and formed the technical manuscripts that underpinned the subsequent consensus meeting, held as the XVII European Workshop in Periodontology in La Granja de San Ildefonso Segovia, Spain, on 7–9 November 2021. The PICO(S) format for systematic reviews provides focused questions to help guide objective outcomes and consists of:

- P – population
- I – intervention/s
- C – comparison/s
- O – outcome/s
- S – study design.

The systematic reviews, once completed, were submitted for peer review and an initial guideline draft with various statements/recommendations was produced by the workshop committee.

As part of the guideline, each statement/recommendation is accompanied by the following information:

- The supporting literature used to arrive at the statement (in most cases these were the systematic reviews produced specifically for the workshop)
- The quality of the evidence from the systematic review
- The grade of recommendation which was categorised into either strong recommendations, recommendations, or open recommendations (Table 2)^7^

**Table 2:** Strength of recommendations grading scheme (Association of the Scientific Medical Societies in Germany – Standing Guidelines Commission, 2023)^7^

**Grade of recommendation**	**Description**	**Syntax**	**Factors influencing recommendation**
A	Strong recommendation	We recommend We recommend not to	Quality of evidence Consistency of study results Magnitude of effect Balance of benefit and harm Ethical, legal and economic considerations Patient preferences
B	Recommendation	We suggest to We suggest not to	
0	Open recommendation	May be considered	
*Unclear	If the group felt evidence was unclear to support a recommendation, statements were formulated including the need for further research.
- The strength of consensus (Table 3).^7^ Participants who voted were required to declare any potential conflicts and would abstain from voting where there was such declaration of interest.

**Table 3:** Strength of consensus determination scheme (Association of the Scientific Medical Societies in Germany – Standing Guidelines Commission, 2023)^7^

**Strength of consensus**	**Definition**
Unanimous consensus	Agreement of 100% of participants
Strong consensus	Agreement of >95% of participants
Consensus	Agreement of 75–95% of participants
Simple majority	Agreement of 50–74% of participants
No consensus	Agreement of <50% of participants

Particular to the EFP Stage IV Guideline, the consensus development conference was held as part of the 17^th^ European Workshop in November 2021 in Spain utilising 13 systematic reviews to provide the basis for an initial draft guideline.

A total of four working groups, which addressed various elements of rehabilitation of periodontitis patients as well as the long-term outcomes and impact of periodontitis treatment, formulated recommendations:

- Group 1 – (chairs: Mariano Sanz and Soren Jepsen): treatment of pathological tooth migration in Stage IV periodontitis patients
- Group 2 – (chairs: Moritz Kebschull and Anton Sculean): treatment of tooth loss/masticatory dysfunction/bite collapse in Stage IV periodontitis patients – partial edentulism amenable for partial rehabilitation
- Group 3 – (chairs: Maurizio Tonetti and Tord Berglundh): treatment of tooth loss/masticatory dysfunction/bite collapse in Stage IV periodontitis patients with terminal dentition only amenable for full-arch rehabilitation
- Group 4 – (chairs: Iain Chapple and David Herrera): long-term outcomes and impact of treatment in Stage IV periodontitis patients.

Due to the breadth of dentistry covered by Stage IV periodontitis, and to simplify the guideline, recommendations were presented as specific case types.

This article therefore summarises these recommendations in the following case types and sections:

- Overall strategy for the management of stage IV periodontitis patients
- Case type 1: the patient with tooth hypermobility that can be corrected without tooth replacement
- Case type 2: the patient with pathological tooth migration, characterised by tooth elongation, drifting and flaring, which is amenable to orthodontic correction
- Case type 3: partially edentulous patients who can be prosthetically restored without full-arch rehabilitation
- Case type 4: partially edentulous patients with a dentition that needs full-arch rehabilitation, either tooth or implant-supported/retained
- Long-term outcomes of treatment in Stage IV periodontitis patients
- Impact of periodontal treatment on systemic health and quality of life.

## UK implementation

In October 2022 the BSP carried out an adolopment process (in a similar fashion to the Stage I–III Guideline)^8^ to ensure the recommendations were pertinent to the UK healthcare system. The UK implementation process followed the ‘GRADE-Adolopment' approach (Schünemann *et al.* 2017). This involves an update of all systematic reviews by the original authors to ensure that new guidelines/evidence had not been published since the original reviews. Furthermore, UK-relevant scientific societies/organisations provided representation for the implementation and, most notably, the BSP patient forum, which provided a valuable patient voice.

A moderated plenary session including all participants of the working groups with a formalised consensus process was then undertaken leading to either:

- Adoption (unmodified acceptance of the recommendation)
- Adaptation (acceptance with modification of the recommendation)
- *De novo* recommendations (formulation of new recommendation if significant new evidence had come to light).

The final BSP adolopment document is titled ‘UK implementation of “Treatment of Stage IV Periodontitis: The EFP S3-Level Clinical Practice Guideline” – rehabilitation of severe periodontitis patients', due to the fact that Stage IV periodontitis from the 2017 World Workshop Classification corresponds to complicating factors rather than bone loss in the apical third of the tooth root as in the BSP implementation of the classification.^5^^,^^6^

## Overall strategy for the management of Stage IV periodontitis patients

Rehabilitation of the periodontitis patient does not follow a specific recipe and requires education of the patient and presentation of various treatment options alongside their benefits/risks as part of the informed consent process. A structured approach which includes treatment of the inflammatory disease is necessary, although once a patient has commenced cause-related therapy it is possible for a restorative plan to run alongside this treatment, e.g., management of occlusal factors or delivery of interim restorations.

**Fig. 1:**
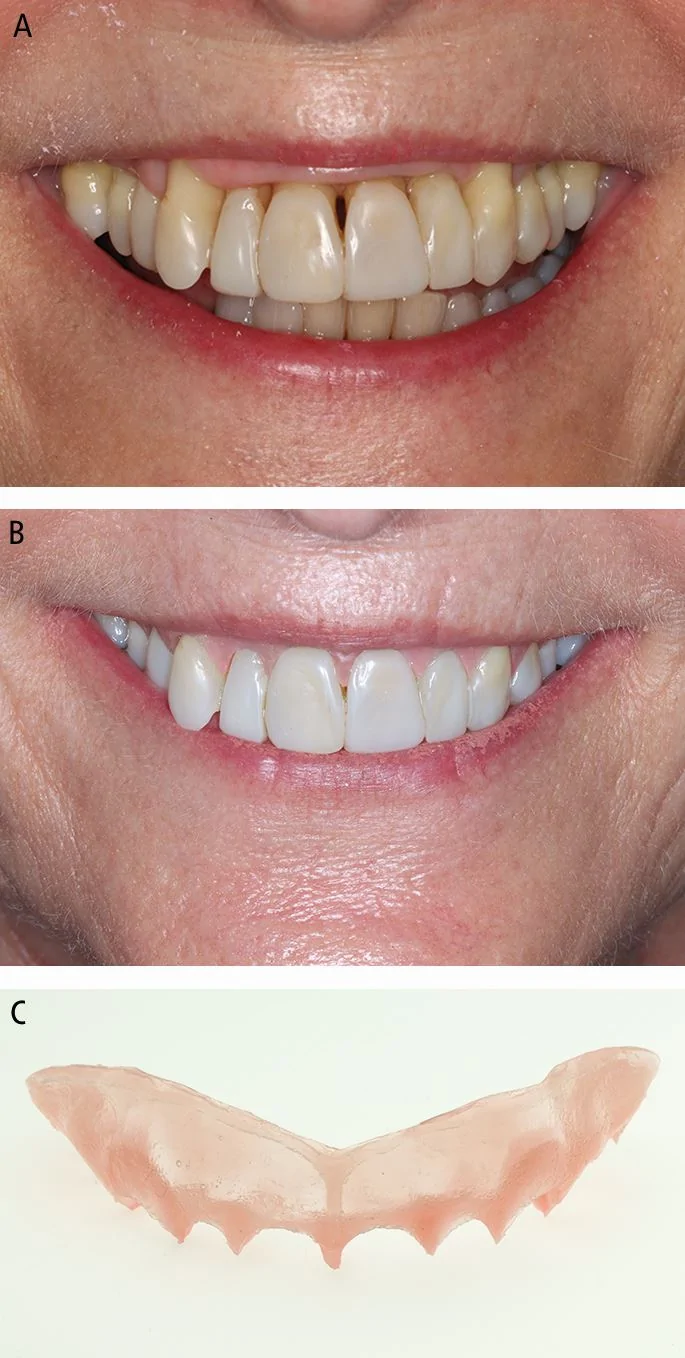
(A, B, C) Gingival veneer demonstrating masking of gingival recession and black triangles following periodontal therapy. Treatment by Dr Devan S. Raindi (specialist periodontist) and laboratory work by Sara Mohammed (Fine Art Dental Studio, Birmingham)

As part of the guideline it is strongly recommended that preservation of teeth is a first line treatment strategy including teeth of a questionable prognosis (Table 4). Whilst restorative therapy aims to restore the function and aesthetics of the dentition, clinicians should first consider simple measures that promote tooth retention alongside those aims. This may include the use of direct/indirect tooth restorations as well as the use of a gingival epithesis (veneer) to improve cosmetics (Fig. 1).

**Table 4:** European Federation of Periodontology Guidelines recommendations for the overall strategy for the management of Stage IV periodontitis patients

**Original EFP recommendations/statements**	**BSP implementation**
Stage IV periodontitis can be successfully managed with the combination of periodontal therapy, appropriate rehabilitation of function and improvement of aesthetics/quality of life, and supportive periodontal care (expert consensus-based **statement**)	Adopted
We **recommend** tooth retention to be the first line of treatment strategy in the rehabilitation of Stage IV periodontitis patients	Adopted
In patients with stage IV periodontitis, we **suggest** preserving the integrity of the dental arch by avoiding extractions, if teeth can be retained	Adopted
In patients with Stage IV periodontitis requesting improvement of aesthetics, phonetics, masticatory function and/or patient well-being, direct and indirect tooth restorations and/or epitheses **may be considered**	Adopted
BSP, British Society of Periodontology; EFP, European Federation of Periodontology

## Case type 1: the patient with tooth hypermobility that can be corrected without tooth replacement

**Fig. 2:**
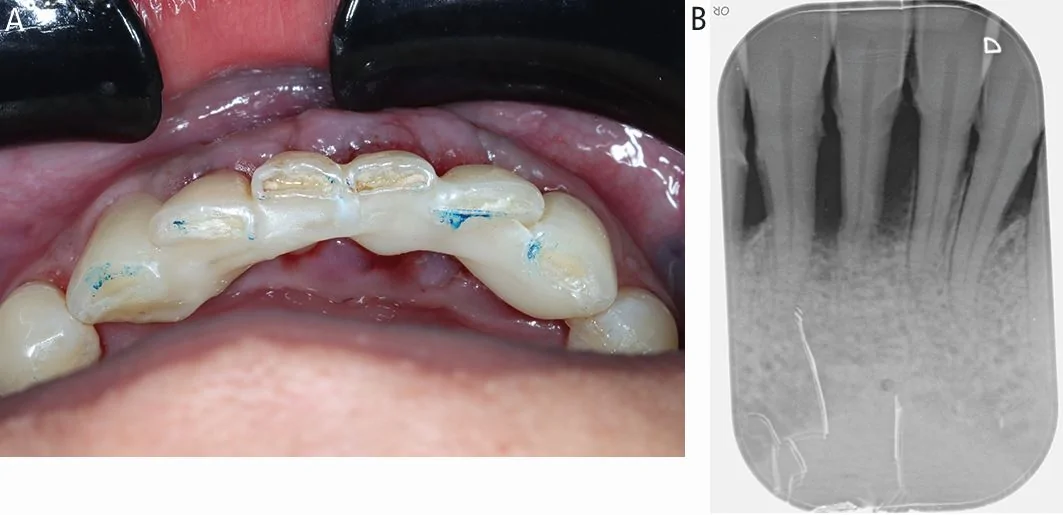
(A, B) Placement of a fibre-reinforced composite splint around the lower incisors with markings assessing the occlusion. Whilst these are bulkier and less cleansable than a wire/composite splint they may be useful in cases where the longer-term prognosis of the tooth is questionable and facilitates future root resection and retention of the crown in the form of an autogenous bridge if appropriate

The use of tooth splints or occlusal adjustment may be considered for the hypermobile tooth with recommendations being open in nature, leaving the final decision to the clinician and patient, based upon the individual case. Figure 2 highlights a case using a fibre-reinforced composite splint for guarded prognosis lower incisor teeth. Both treatment modalities are generally used with the aim of improving patient comfort and facilitating periodontal therapy including patient home care. Table 5 highlights the recommendations associated with the management of patients with tooth hypermobility.

**Table 5:** European Federation of Periodontology Guidelines recommendations for the management of patients with tooth hypermobility that can be corrected without tooth replacement

**Original EFP recommendations**	**BSP implementation**
In patients with stage IV periodontitis, temporary splinting and/or limited selective occlusal adjustment of hypermobile teeth **may be considered** during all steps of periodontal therapy (but particularly during step one treatment) to increase patient comfort and enable/facilitate periodontal therapy	Adopted
In patients with Stage IV periodontitis who do not require tooth replacement but show persistent hypermobility or increasing mobility after successful completion of periodontal therapy, long-term tooth splinting **may be considered** to improve patient comfort	Adopted
BSP, British Society of Periodontology; EFP, European Federation of Periodontology	

## Case type 2: the patient with pathological tooth migration, characterised by tooth elongation, drifting and flaring, which is amenable to orthodontic correction

The complicating factors that arise from advanced periodontitis can lead to functional and aesthetic compromise. Orthodontic therapy can be a valuable treatment modality in rehabilitating the periodontitis patient who experiences such complications. Table 6 highlights the recommendations associated with the management of patients where orthodontic correction is considered as part of treatment.

**Table 6:** European Federation of Periodontology Guidelines recommendations for the patient with pathological tooth migration that can be corrected with orthodontic therapy

**Original EFP recommendations**	**BSP implementation**
In successfully treated Stage IV periodontitis patients in need of orthodontic therapy, we **suggest** undertaking OT based on evidence that: it does not significantly affect periodontal outcomes (probing pocket depth-PPD and clinical attachment levels-CAL) it does not significantly affect gingival inflammation (bleeding on probing—BOP) and gingival recession it does not lead to a significant increase in root resorption	Adopted with editorial change to recommendation 3 to now state ‘it may not lead to an elevated risk of root resorption'
In successfully treated Stage IV periodontitis patients in need of orthodontic therapy, we **recommend** starting OT once the endpoints of periodontal therapy have been achieved [no sites with PPD = 5 mm and BOP and no sites with PPD ≥6 mm (Sanz, Herrera, *et al.* 2020)]	Adapted to follow the endpoints of therapy which are defined in the BSP Implementation of the Stage I–III Guideline (West *et al.* 2021)
In Stage IV periodontitis patients with pathological tooth migration, we suggest undertaking orthodontic therapy once the endpoints of periodontal therapy have been reached, based on the evidence that this therapy: does not significantly affect periodontal outcomes [CAL, PPD, and radiographic bone levels (RBL)] seems to reduce gingival inflammation (BOP) does not significantly alter gingival margin levels seems to improve interdental papilla height does not significantly affect root resorption and seems to reduce tooth mobility	Adapted for recommendation 5 to now state ‘does not significantly affect root resorption' All other recommendations adopted
In stage IV periodontitis patients with tilted molars orthodontic therapy **may be considered**, although there is a lack of evidence on its possible effect on periodontal outcomes	Adopted
In stage IV periodontitis patients where intra-bony defects have been treated following the recommendations of the clinical practice guideline ([Sanz, Herrera, *et al.*,2020] using the appropriate regenerative interventions): We **recommend** undertaking OT based on the evidence that the combined treatment significantly improves periodontal outcomes (increased CAL gain, PPD reduction and RBL gain) and significantly reduces gingival inflammation (BOP) We **suggest** not to wait for a prolonged healing period after the regenerative intervention, before initiating OT, since there is evidence that a short (one month) and a prolonged (six months) period between periodontal/regenerative and OT result in comparable outcomes	Adapted for recommendation 2 to now state (downgraded): *A short (one month) healing period between periodontal/regenerative treatment and orthodontics* ***may be considered***. *There is weak evidence that a shorter (one month) and prolonged (six months) period between periodontal/regenerative and orthodontic treatment results in comparable outcomes* Recommendation 1 is adopted
In patients with severe periodontitis (stage IV or equivalent) with indication of OT to maintain/improve periodontal stability: We **suggest** using fixed rather than removable appliances Use of fiberotomy as an adjunct to orthodontic tooth movement **may be considered** to improve periodontal outcomes Use of skeletal anchorage devices (implants or temporary anchorage devices—micro-screws or mini-plates) **may be considered** to enhance orthodontic tooth movement	Adapted to: *In patients with severe periodontitis (stage IV or equivalent) with indication of OT to maintain/improve periodontal stability:* *The use of either fixed or removable appliances may be considered* *There is a lack of evidence to endorse the use of fiberotomy to improve periodontal outcomes as an adjunct to orthodontic tooth movement* *The use of skeletal anchorage devices (implants or TADs – micro-screws or miniplates) may be considered to enhance orthodontic tooth movement*
We **recommend** that during OT the patient's periodontal status is closely monitored and managed, ideally at each orthodontic appointment. If signs of periodontitis recurrence are detected, active OT should be interrupted, and the affected teeth should be maintained passively, while rendering proper periodontal treatment and oral hygiene reinforcement. Once periodontal health/stability has been re-established, OT can be re-instituted We **recommend** that after completion of OT, life-long supportive periodontal care and life-long orthodontic retention are provided, tailored to the individual needs/risk profile of the patient	Adapted to: *We* ***recommend*** *that the periodontal status is monitored regularly during OT. If signs of recurrence of periodontitis are detected, active orthodontic forces should be withdrawn, while periodontal treatment and oral hygiene reinforcement are instituted. Once periodontal health/stability has been re-established, active orthodontic forces can be re-instituted where indicated*
We **recommend** that an appropriately designed permanent fixed passive retention (with or without additional removable retention) is used after the completion of orthodontic therapy We also **recommend** that patients enter a life-long supporting protocol to identify early retainer failures and/or undesired tooth movements	Adapted to: *We* ***recommend*** *that appropriately designed life-long passive retention (involving fixed and/or removable designs) is used after the completion of orthodontic treatment. The specific approach should be tailored based on a range of factors including the likely potential for post-treatment change, presence of differential tooth mobility, cleanability, cost and patient preferences*
BOP, bleeding on probing; BSP, British Society of Periodontology; EFP, European Federation of Periodontology; CAL, clinical attachment level; OT, orthodontic therapy; PPD, probing pocket depth; RBL, radiographic bone level; TAD, temporary anchorage device	

Recommendations clearly state that endpoints of periodontal therapy should be achieved, i.e., the patient should be stable prior to commencing orthodontic tooth movement. In the stable and well-maintained periodontitis patient there is little evidence to suggest orthodontic therapy is detrimental to periodontal clinical parameters and it may even improve interdental papilla height.

The approach to orthodontic therapy has also been considered with both fixed and removable appliances deemed suitable for tooth movement. The use of skeletal anchorage devices may assist in meeting anchorage requirements and reduce the forces required on teeth with a reduced albeit healthy periodontium.

**Fig. 3:**
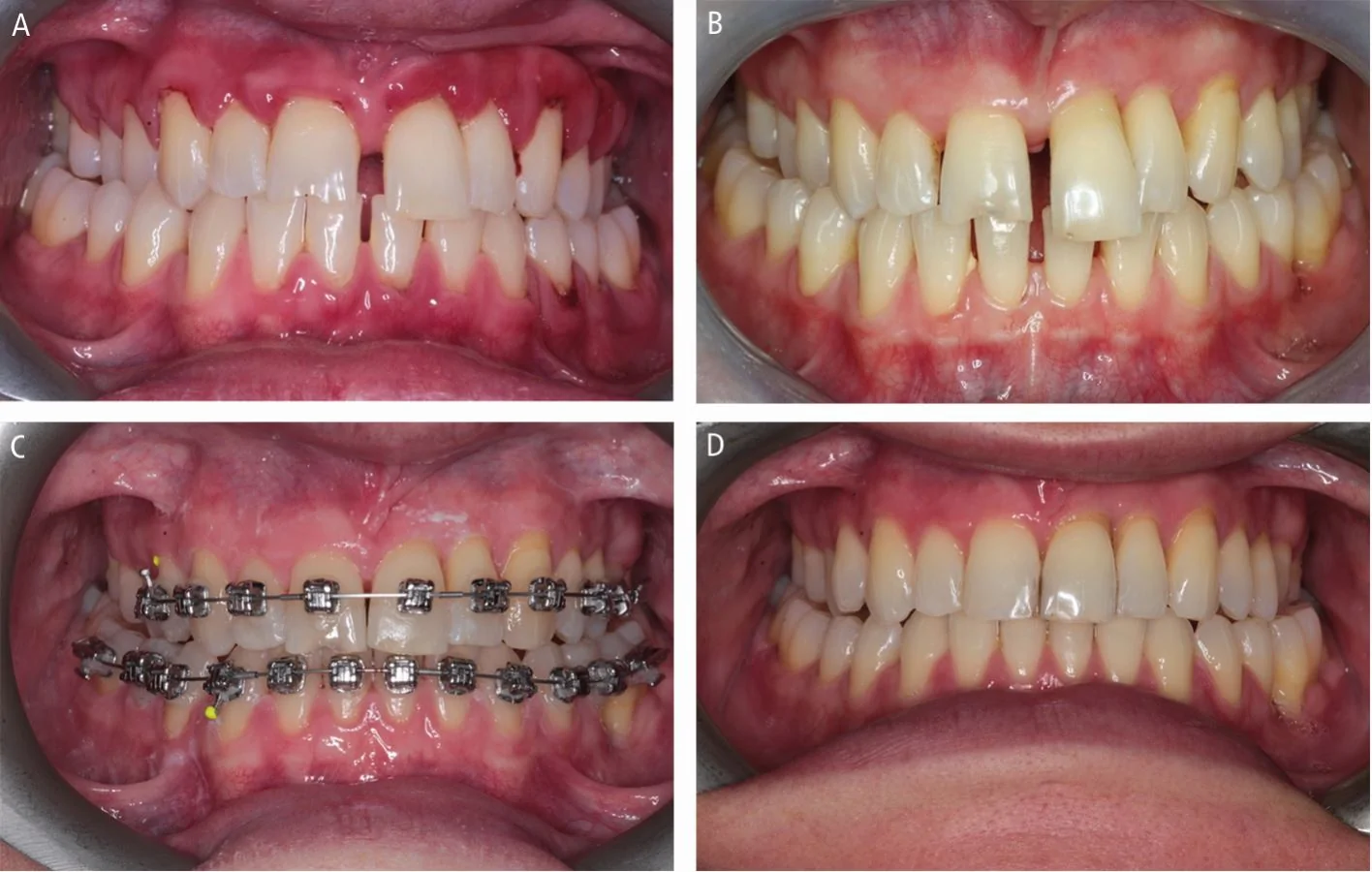
(A, B, C, D) Drifting and overeruption due to periodontitis. Following control of periodontitis, fixed appliances were used to align the teeth and intrude 21 alongside closely monitored supportive therapy. The final result demonstrates closure of the midline diastema and improved papilla infill between the upper anterior teeth. Periodontal treatment completed by Dr Devan S. Raindi (specialist periodontist) and orthodontic treatment by Dr Gursh Bajwa (dentist with special interest in orthodontics)

It is imperative that during orthodontic therapy, periodontitis patients have their periodontium closely monitored for signs of relapse. If recurrence is identified, orthodontic tooth movement should be stopped, the teeth maintained passively, and the periodontitis treated. Once health has been re-established orthodontic tooth movement can continue. At the end of orthodontic treatment, periodontitis patients should remain in life-long supportive periodontal care on an appropriate interval frequency based upon their risk profile. Figure 3 highlights a case where orthodontic tooth movement was completed with a fixed appliance following treatment of periodontitis in a young adult.

**Fig. 4:**
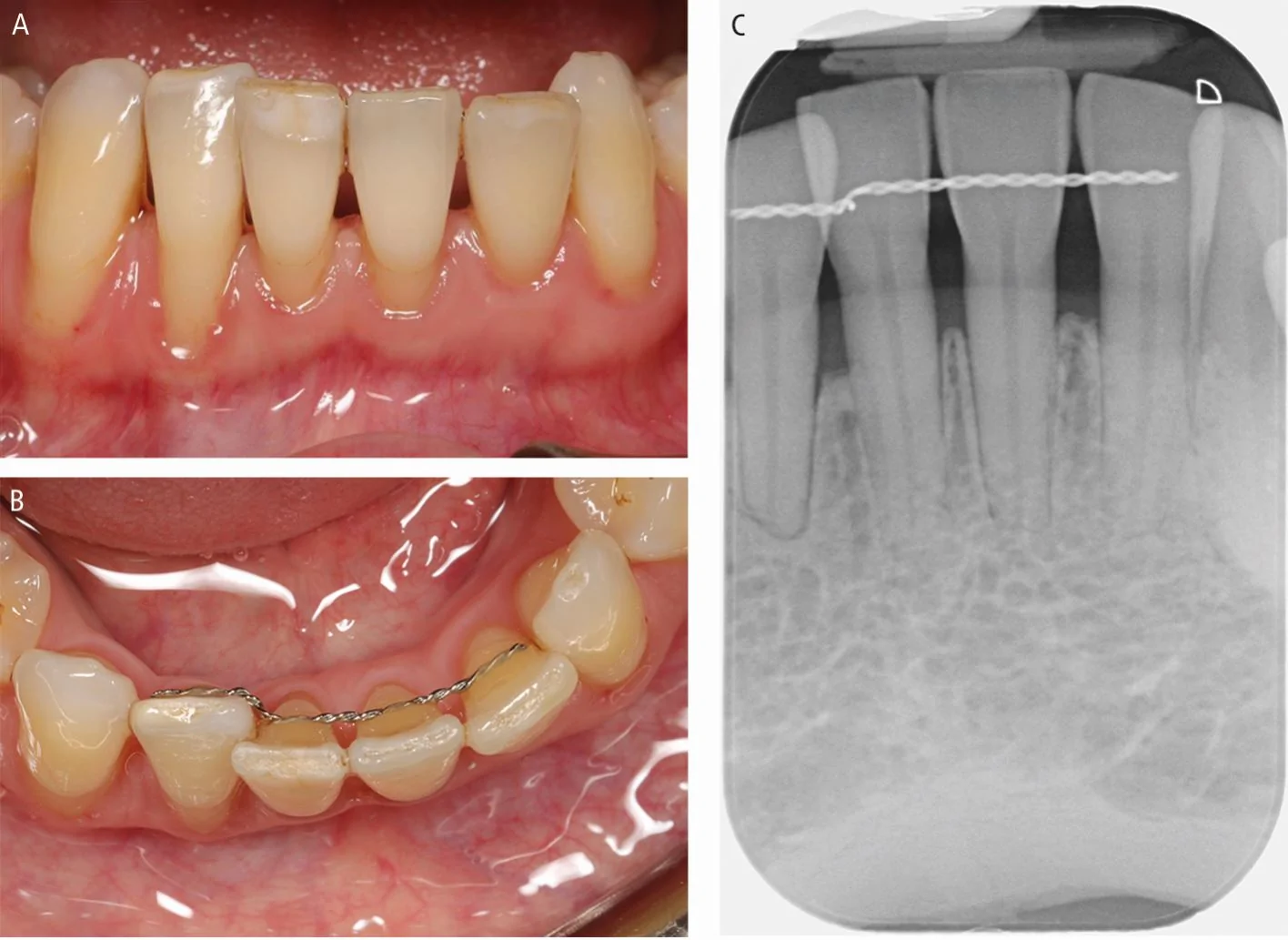
(A, B, C) Patient presenting with progressive gingival recession affecting the lower incisor teeth and lower right canine. Occlusal view demonstrates a de-bonded twisted wire retainer (placed 14 years ago) and evidence of a torquing effect on the roots of the incisor teeth (buccally on 42, 43 and lingually on 41)

Life-long supportive maintenance should not only monitor the patient's periodontal status but also the integrity of any fixed passive retention, identifying early debonding as well as unwanted tooth movements (a phenomenon known as ‘wire syndrome') which may predispose to extensive recession defects (Figure 4).^9^

## Overall strategy for the management of case types 3 and 4

Rehabilitation of the dentition is specific to the individual case and wishes of the patient. General principles help guide our decisions and have been considered as part of the guideline document with overarching recommendations highlighted in Table 7.

**Table 7:** European Federation of Periodontology Guidelines recommendations for the overall strategy for management of case types 3 and 4

**Original EFP recommendations**	**BSP implementation**
We **recommend** identifying the restorative needs of partially edentulous stage IV periodontitis patients based on the pattern of tooth loss, individual functional and aesthetic needs, patient comfort and prognostic factors. The level of function and design of the rehabilitation should be compatible with case stability over time	Adopted
We **recommend** placing an interim dental prosthesis, if required, early during periodontal therapy, but only after establishing adequate oral hygiene	Adapted to: *We* ***recommend*** *placing an interim dental prosthesis, if required, early during periodontal therapy, but generally only after establishing adequate oral hygiene*
We **recommend** designing the dental prostheses to allow optimal self-performed oral hygiene measures and professional mechanical plaque removal We **recommend** delivery of the definitive prosthesis after a final evaluation of the maintainability and prognosis of abutment teeth/implants	Adapted to: *We* ***recommend*** *designing the dental prostheses to allow optimal self-performed oral hygiene measures, monitoring of periodontal and peri-implant tissues and professional mechanical plaque removal.* *We* ***recommend*** *delivery of the definitive prosthesis after a final evaluation of the maintainability and prognosis of abutment teeth/implants*
When dental implants are considered in the rehabilitation of stage IV periodontitis patients, we **recommend** verifying: Absence of contraindications to surgery Hard and soft tissue dimensions The potential need for soft-/hard-tissue augmentation	Adapted to: *When dental implants are considered in the rehabilitation of stage IV periodontitis patients, we* ***recommend*** *verifying* *(1) absence of contraindications to surgery, (2)* ***quality and quantity*** *of hard and soft-tissues, and (3) the potential need for soft/hard tissue augmentation*
When dental implants are considered in the rehabilitation of stage IV periodontitis patients, we **recommend** that information on the increased risk for peri-implantitis and implant loss should be provided	Adopted
Due to the risk of tooth loss and prosthesis failure, we **suggest** avoiding combined tooth/ implant-supported fixed partial dental prostheses if alternatives are feasible	Adapted to: *Due to the risk of tooth loss and prosthesis failure, we* ***suggest*** *avoiding combined tooth/implant- supported fixed partial dental prostheses*
BSP, British Society of Periodontology; EFP, European Federation of Periodontology	

**Fig. 5:**
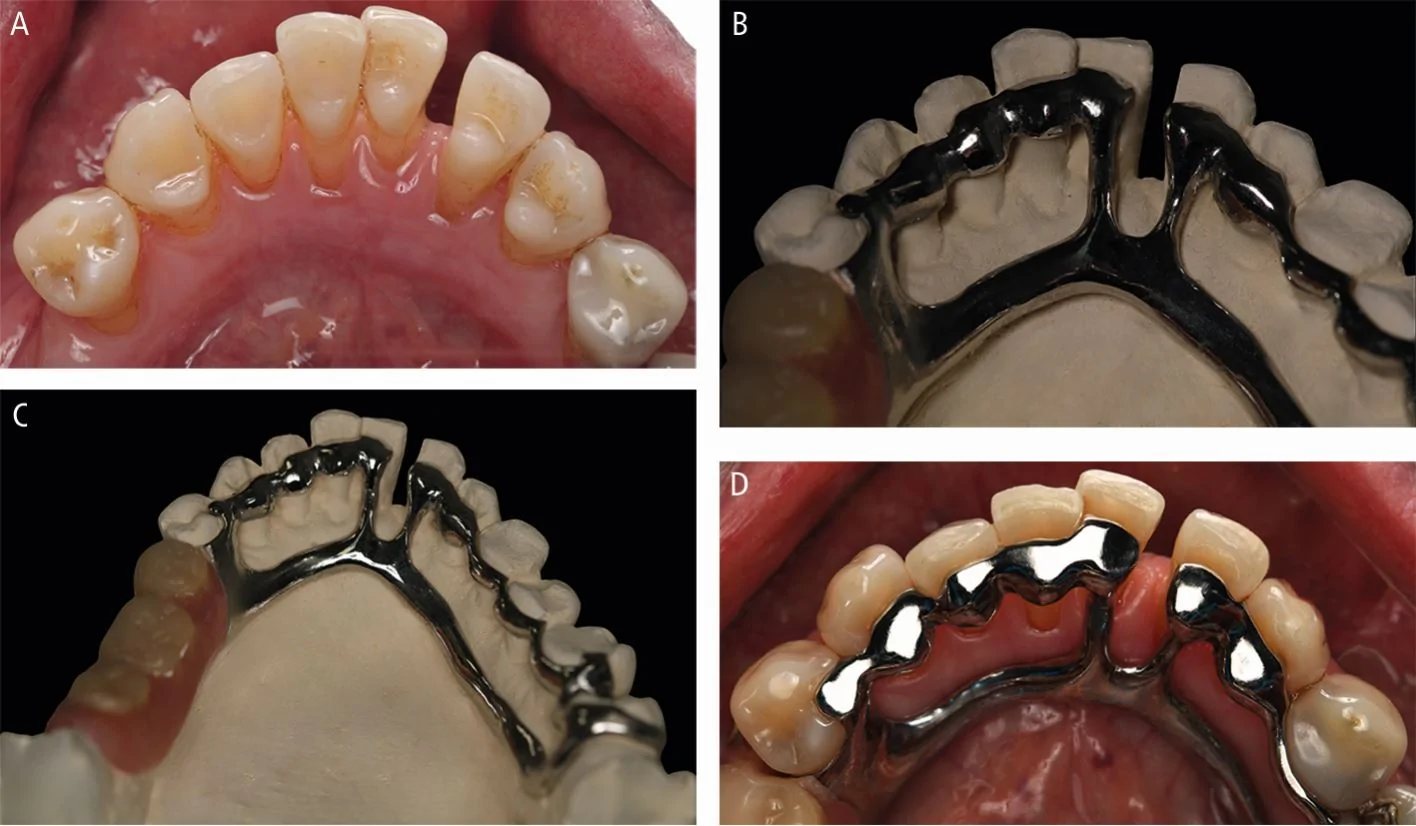
(A) Pre-operative view showing stable periodontal status. (B, C) Master cast views of the scalloped ‘Scandinavian' framework designed to optimise hygiene access. (D) Post-operative view in situ demonstrating deliberate gingival clearance to facilitate long-term periodontal maintenance. Treatment by Dr Sandeep Mukar (specialist prosthodontist) and laboratory work by Chris Wibberley (CW Dentures)

**Fig. 6:**
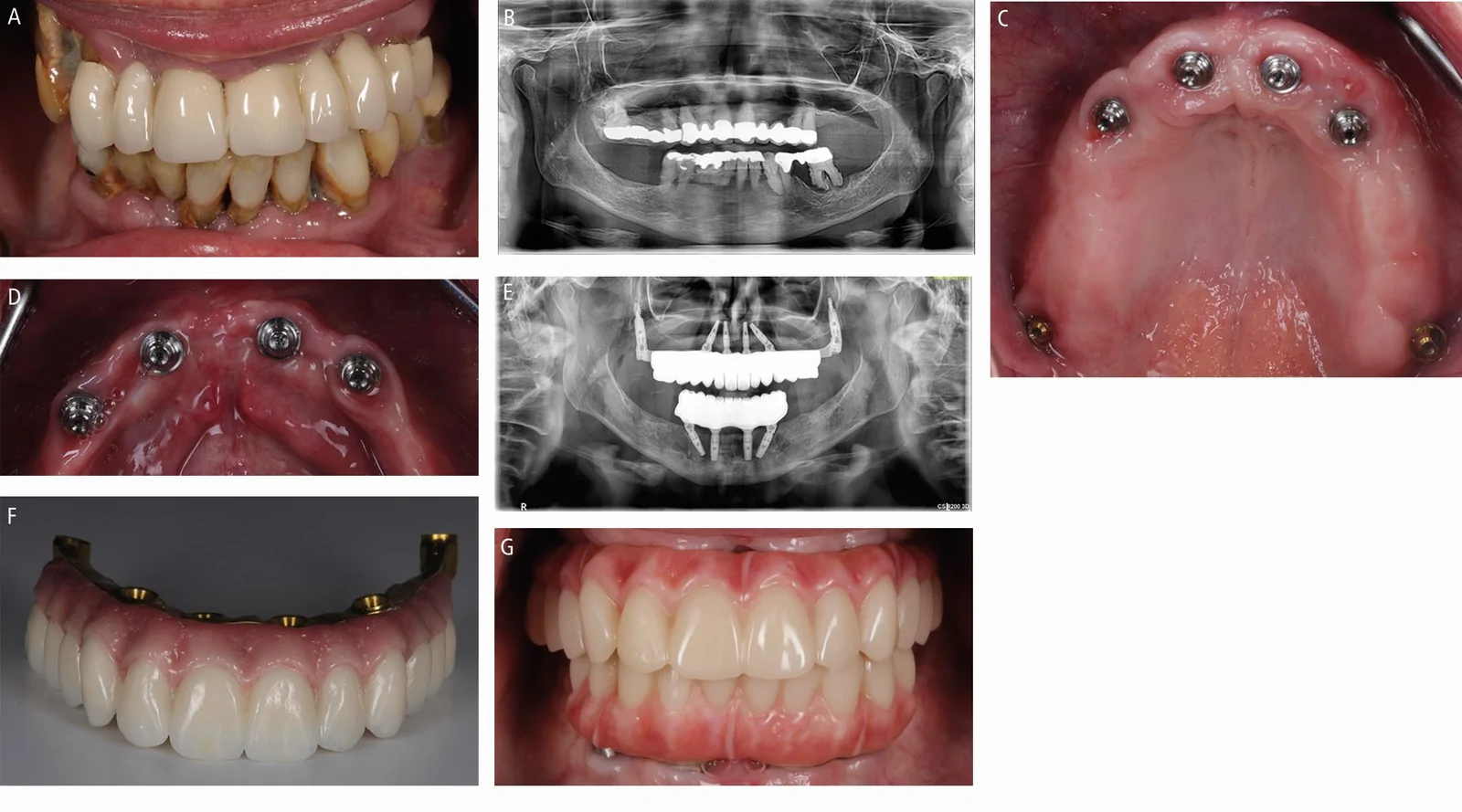
(A) Terminal dentition and bite collapse in a previously heavily restored dentition. (B) Pre-operative orthopantomogram (OPG) demonstrating advanced bone loss. (C) Upper arch treated with six implants. (D) Lower arch treated with four implants. (E) Post-operative OPG demonstrating appropriate spread of implants to allow for full arch fixed prosthesis. (F) Maxillary prosthesis. (G) Post-operative frontal view. Implants placed Dentsply Primetaper EV and Noris Pteryfit. Treating clinician Dr Ricky Bhopal (specialist prosthodontist) and laboratory work by Kevin Armstrong

Of note, interim restorations are a valuable step in restoring the dentition but should only be considered once Step 1 of periodontitis treatment has been initiated, i.e., the patient demonstrates an appropriate standard of oral hygiene. Dental prostheses (fixed or removable) should be made cleansable for both patients and professionals (Fig. 5, Fig. 6).

Dental implants remain a valuable method for replacing missing teeth and rehabilitating the dentition but in the periodontitis patient it is important that the increased risk for peri-implantitis and implant loss is discussed as part of informed consent. Furthermore, treatment of periodontitis should be commenced before dental implants are placed, as unstable periodontitis remains a key risk factor for biological complications. An S3-Level Guideline for the Prevention and Treatment of Peri-Implant Diseases has also been produced (and adoloped by the BSP), providing more guidance on this.^10^^,^^11^

The various restorative recommendations for case type 3 are demonstrated in Table 8 and have been split into management of either tooth-delimited gaps or free-end edentulism.

**Table 8:** European Federation of Periodontology Clinical Guidelines recommendation for the management of case type 3 (restoration of the partially edentulous patient)

**Original EFP recommendations**	**BSP implementation**
**Tooth-delimited gaps**	
In partially edentulous Stage IV periodontitis patients with tooth-delimited edentulous spaces, different options (namely tooth-supported fixed dental prostheses, implant-supported fixed dental prostheses, removable dental prostheses, or no prosthetic rehabilitation) **may be considered**	Adopted
We suggest using tooth-supported fixed dental prostheses in patients with Stage IV periodontitis when abutment teeth are periodontally maintainable and restorable Resin-bonded fixed dental prostheses **may be considered** in certain circumstances (e.g., small tooth-delimited edentulous spaces) We **suggest not to** use resin-bonded fixed dental prostheses for large tooth-delimited edentulous spaces	Adapted to: *We* ***suggest*** *using tooth-supported fixed dental prostheses in patients with stage IV periodontitis when abutment teeth are periodontally maintainable and restorable* *Resin-bonded fixed dental prostheses* ***may be considered*** *in certain circumstances (e.g., small tooth delimited edentulous spaces* *and good quality of the abutment tooth/teeth)* *We suggest not to use resin-bonded fixed dental prostheses for large tooth-delimited edentulous spaces*
We **suggest** using implant-supported fixed dental prostheses when abutment teeth are not periodontally maintainable and restorable	Adopted
Metal-based frame removable dental prostheses **may be considered** as transitional or definitive treatment options when a fixed solution is not a consideration	Adopted
**Unilateral or bilateral posterior free-end edentulism**	
For the rehabilitation of partially edentulous Stage IV periodontitis patients with free-end situations, different options (namely shortened dental arch, implant-supported restorations or removable dental prostheses) **may be considered**	Adopted
In Stage IV periodontitis patients with a shortened dental arch with sufficient occluding/masticatory units (e.g., from second premolar to second premolar, no evident risk for flaring or tooth elongation and adequate patient comfort) no tooth replacement may be considered in the free-end situation	Adopted
In Stage IV periodontitis patients with free-end situations who require additional occluding units, we suggest implant-supported fixed dental prostheses When implants are not an option, we **suggest** removable dental prostheses with a metal-based framework	Adapted to: *In Stage IV periodontitis patients with free-end situations who require additional occluding units, we suggest implant-supported fixed dental prostheses. When only an extra occlusal unit is needed, tooth-supported fixed cantilever FDP* ***may be considered*** *to establish a SDA* *When implants are not an option, we* ***suggest*** *removable dental prostheses with a metal-based framework*
BSP, British Society of Periodontology; EFP, European Federation of Periodontology; FDP, fixed dental prosthesis; SDA, shortened dental arch	

Where full-arch rehabilitation is deemed the most appropriate means of restoration, recommendations are summarised in Table 9 and Table 10 and considered as either tooth-supported full arch fixed prostheses or implant-retained full arch rehabilitation.

**Table 9:** European Federation of Periodontology Guidelines recommendation for the management of case type 4 (cases requiring full arch rehabilitation)

**Original EFP recommendations**	**BSP implementation**
In patients with periodontitis Stage IV with a sufficient number (≥four abutment teeth) of periodontally maintainable, bilaterally distributed and restorable teeth in the maxilla and/or mandible, we **suggest** a tooth-supported full-arch fixed dental prosthesis	**Adapted to:** *In patients with periodontitis Stage IV with a sufficient number (≥four abutment teeth) of periodontally maintainable, bilaterally distributed and restorable teeth in the maxilla and/or mandible, a tooth-supported full-arch fixed dental prosthesis may be considered*
In periodontitis Stage IV patients with an insufficient number/distribution of periodontally maintainable teeth to support a tooth-supported full-arch fixed dental prosthesis, a tooth-supported full-arch removable dental prosthesis (overdenture) **may be considered**	Adopted
In periodontitis Stage IV patients in whom tooth preservation was deemed impossible, and a sufficient number (≥four) of bilaterally distributed and adequately sized dental implants are planned in the maxilla and/or mandible, we **suggest** an implant-supported full-arch fixed dental prosthesis	Adopted
In periodontitis Stage IV patients in whom tooth preservation was deemed impossible and adequately sized dental implants can be used, albeit not in sufficient number and/or adequate position to support a full-arch fixed dental prosthesis, an implant-supported full-arch removable dental prosthesis (overdenture) **may be considered**	Adopted
BSP, British Society of Periodontology; EFP, European Federation of Periodontology

**Table 10:** European Federation of Periodontology Guidelines recommendations for the long-term outcomes of periodontal therapy in periodontitis patients

**Original EFP recommendations/statements**	**BSP implementation**
We **recommend** provision of and adherence to regular professionally administered SPC to reduce tooth loss in the long term (≥five years) We **recommend** that SPC should initially be provided at three-monthly intervals. Medium- to long-term frequency should be personalised to each individual patient, taking into account clinical and behavioural circumstances	Adapted to: 1) We recommend provision of, and adherence to, regular professionally administered SPC to reduce tooth loss in the long term (≥five years) 2) We recommend that SPC should initially be provided at three-monthly intervals. Medium to long- term frequency should be personalised to each individual patient, taking into account clinical and behavioural circumstances with a maximum interval of 12 months
In patients with periodontitis, the presence of residual probing pocket depths (≥5 mm) following active therapy increases the risk of disease recurrence/progression, even though the patient is enrolled in SPC	Adopted
We **recommend** that recall intervals for supportive periodontal care (SPC) should be guided by patients' risk profile as determined by individual risk factors (e.g., smoking, hyperglycaemia) and disease-associated clinical measures (such as pocket depths and bleeding on probing)	Adopted
We **recommend** a number of important components when designing an SPC programme, including: Specific interventions include interview, assessment, evaluation, practical intervention and planning Delivery by a variety of oral healthcare professionals, under the supervision of a suitably trained general dentist or a specialist, as appropriate to case complexity Clear two-way communication between the oral healthcare team and the patient, and between healthcare professionals (medical or dental)	Adopted
We **suggest not to** use adjunctive approaches to subgingival instrumentation when treating recurrence of periodontitis during supportive periodontal care	Adopted
There is no evidence of clinical disadvantages to regular long-term SPC, such as gingival recession/clinical attachment loss; however, the possibility of these side effects cannot be excluded based on the evidence reviewed. Patients should be advised of this as part of their informed consent	Adopted
We **suggest** that regular long-term SPC in specialist practice may result in greater periodontal stability and tooth survival when compared with SPC in general practice We **do not know** if long-term SPC is cost-effective when considering direct and indirect costs	Adopted
We **do not know** if long-term SPC impacts upon patient-reported outcomes	Adopted
BSP, British Society of Periodontology; EFP, European Federation of Periodontology; SPC, supportive periodontal care	

## Long-term outcomes of treatment in Stage IV periodontitis patients

Supportive periodontal care (SPC) is an important component of the treatment strategy for any patient with periodontitis (Step 4) irrespective of disease stage and the recommendations in Table 10 are therefore applicable for all forms of periodontitis.

It is important to note that despite our best efforts as clinicians to reach the endpoints of periodontitis and complete stability, there may be cases in the real world where this is not feasible and patients with residual probing pocket depths are enrolled into Step 4 of therapy. A recent systematic review and meta-analysis highlighted endpoints achieved in as few as 1.35% of patients treated for periodontitis based on the definition used.^12^ This does not mean undertreating the disease, however, and if the treating clinician has any doubts about the periodontal status, patients should be referred for specialist opinion. Whilst the endpoints met can be low based on the criteria used, it is also essential to highlight that only approximately 3% of teeth included in the study (323,111 total teeth included) were lost, reinforcing the importance that in treated periodontitis patients placed in regular supportive therapy, tooth loss remains very low.^12^

The components of an SPC visit have been recommended and include:

- Interview: ascertaining any symptoms and taking a comprehensive history including risk factor assessment
- Assessment: taking periodontal clinical measures, for example plaque/bleeding scores and probing pocket depths
- Evaluation/care planning: of intervention needs (e.g., risk factor management, oral hygiene and re-treatment)
- Communicating: findings to patient and consent for treatment
- Practical intervention: oral hygiene advice, supra/subgingival professional mechanical plaque removal
- Planning/recall interval: recall interval for next SPC visit. Recalls should initially be set at three-monthly intervals and then individually titrated according to patient stability, risk, preference and perceived clinical need. Impact of periodontal treatment on systemic health and quality of life.

Research exploring the impact of periodontitis treatment on chronic non-communicable diseases (NCDs), such as diabetes and cardiovascular disease, has increased significantly in recent years. Recommendations, however, remain unclear on whether periodontitis improves ‘hard' outcomes of systemic NCDs (e.g., myocardial infarction, death) or improves levels of biomarkers of systemic inflammation in healthy individuals. The reason for this is not due to evidence demonstrating lack of effect, but rather the lack of sufficient evidence for which further research has been recommended.

On the other hand, in individuals with a co-morbid NCD, it is suggested that treatment of periodontitis is performed to reduce systemic inflammation. New evidence in the form of a Cochrane systematic review has resulted in a *de novo* recommendation to treat periodontitis in people with diabetes due to the reductions in HbA1c levels that are achieved (Table 11).^13^ This systematic review and meta-analysis found a mean reduction of 4.7 mmol/mol (0.43)% HbA1c at 3 to 4 months following treatment of periodontitis and highlights the nature of the S3 guideline as a ‘living document' open to update based on new research.

**Table 11:** European Federation of Periodontology Guidelines recommendations for the impact of periodontal treatment on systemic health and quality of life

**Original EFP recommendations**	**BSP implementation**
Treatment of periodontitis **may improve** levels of biomarkers of systemic inflammation and cardio-metabolic risk in people with no reported systemic co-morbidity	Adopted
It is **currently unclear** if the treatment of periodontitis improves ‘hard' outcomes or complications of systemic NCDs in patients with periodontitis with a co-morbid NCD	Adopted
We **suggest** that treatment of periodontitis is performed to reduce systemic inflammation, to reduce cardiovascular risk profile and to improve metabolic control in patients with co-morbid NCDs; however, treatment protocols should include careful consideration of the general health status of the patient (e.g., quadrant vs. full-mouth approach in those with elevated cardiovascular risk)	Adopted with an additional new evidence-based recommendation of: *We* ***recommend*** *that treatment of periodontitis is* ***performed*** *to improve metabolic control in patients with diabetes. Protocols should include careful consideration of the general health status of the patient (e.g., quadrant versus full-mouth approach)*
It is **unclear** whether treatment of periodontitis during pregnancy may reduce pre-term births (<37 weeks) or reduces other adverse pregnancy outcomes	Adopted
We recommend rehabilitation of people with partial edentulism of at least five teeth (including those affected by periodontitis) to improve quality of life Rehabilitation of partial edentulism with tooth-supported, fixed or removable prostheses improves quality of life Rehabilitation of partial edentulism with implant-supported prostheses improves quality of life	Adopted
We **recommend** providing complete conventional removable prostheses to treat fully edentulous patients (including those who have lost teeth due to periodontitis), in one or both arches, to improve quality of life	Adopted
We **suggest** treating fully edentulous people (including those previously affected by periodontitis) with implant-supported full-arch removable dental prosthesis (overdenture), rather than conventional full-arch removable prostheses, to improve quality of life	Adopted
We **recommend** prosthetically treating fully edentulous people in order to improve nutritional status We **do not know** if treating full edentulism is associated with improvement of frailty, cognitive function or other systemic health benefits	Adopted
BSP, British Society of Periodontology; EFP, European Federation of Periodontology; NCD, non-communicable disease	

Both the treatment of periodontitis and rehabilitation of the dentition have also been shown improve quality of life outcome measures in patients demonstrating a positive impact beyond the oral cavity.

## Conclusion

Treating periodontitis and its complications can provide significant improvements in our patient's quality of life as well as their systemic health.

The BSP adolopment of the EFP Guidelines for the Treatment of Stage IV Periodontitis provides an evidence-based guidance for clinicians in the UK to help inform decision-making in the rehabilitation of periodontitis patients.
